# The prevalence of infertility and factors associated with infertility in Ethiopia: Analysis of Ethiopian Demographic and Health Survey (EDHS)

**DOI:** 10.1371/journal.pone.0291912

**Published:** 2023-10-12

**Authors:** Nanati Legese, Abera Kenay Tura, Kedir Teji Roba, Henok Demeke

**Affiliations:** 1 School of Pharmacy, College of Health and Medical Sciences, Haramaya University, Harar, Ethiopia; 2 Department of Obstetrics and Gynecology, University Medical Centre Groningen, University of Groningen, Groningen, The Netherlands; 3 School of Nursing and Midwifery, College of Health and Medical Sciences, Haramaya University, Harar, Ethiopia; Hormozgan University of Medical Sciences, ISLAMIC REPUBLIC OF IRAN

## Abstract

**Background:**

Despite having a high fertility rate, low-resource countries are also home to couples with infertility problems. Although many couples are suffering from the psychological impacts of infertility, its level and determinants are not adequately known. The main objective of this study is to assess the prevalence and factors associated with infertility among couples in Ethiopia using the 2016 Ethiopian Demographic and Health Survey (EDHS) data.

**Method:**

The study employed a cross-sectional study design extracting variables from the 2016 EDHS. The study included all married or cohabitating women aged 15 to 49 years in the Couples Recode (CR) file data set. Weighted samples of 6141 respondents were analyzed. We used Stata 14 software for analyzing the data. The association of selected independent variables with primary, secondary, and total infertility was analyzed using a logistic regression model. We presented the results using an adjusted odds ratio (AOR) with a 95% confidence interval (CI) and a p-value <0.05 as a cut-off point for declaring statistical significance.

**Results:**

The prevalence of infertility in the past 12 months was 24.2% (95% CI: 23.1–25.3%), of which the majority (90.7%) was secondary infertility. Greater than 35 years of age (AOR = 2.45, 95% CI (1.58–3.79)), rural residence (AOR = 1.06, 95% CI (1.01–1.39)), smoking (AOR = 2.29, 95% CI (1.39–3.77)), and <18.5 Body Mass Index (BMI) (AOR = 1.71, 95% CI (1.43–2.04)) were significantly associated with infertility. Conversely, infertility was less likely among women with formal education and better wealth index. Primary infertility was significantly higher among women whose partners drink alcohol (AOR = 1.55; 95% CI 1.06–2.28)) and chew khat (AOR = 1.62; 95% CI (1.12–2.36)). Secondary infertility was significantly higher among women with <18.5 BMI (AOR = 1.59, 95% CI (1.37–1.84)), >30 BMI (AOR = 1.54; 95% CI 1.01–2.35)), and <15 years of age at first birth (AOR = 1.40; 95% CI 1.15–1.69)).

**Conclusion:**

More than one in five couples in Ethiopia has an infertility problem. Both male and female-related factors are associated with infertility. Primary infertility was significantly higher among women whose partner chews khat and drinks alcohol. Secondary infertility was significantly associated with being underweight, obese, smoking, and young age at first birth. Hence, taking action on preventable factors is the most critical treatment approach and will improve the health status of the couples in other ways.

## Introduction

Infertility is the failure to achieve a clinical pregnancy after 12 months or more of regular unprotected sexual intercourse [1]. Infertility is classified into primary and secondary based on the presence or absence of previous pregnancy. Primary infertility is the inability to conceive, while secondary infertility is the inability to bear a child after having an earlier birth [2].

Infertility is a critical issue for couples of childbearing age over the world. Fifteen percent of couples around the world are suffering from infertility, in half of which the man is infertile.

In some parts of sub-Saharan Africa, a 15 to 45% prevalence was reported [3–6]. Infertility has significant negative social impacts on the lives of infertile couples and particularly women, being blamed for the problem [7–12].

Although there is a significant concern about the impacts of infertility, there are no comprehensive epidemiological studies about its risk factors in resource-limited countries [13]. Conventionally, factors such as age, obstetrical history, smoking, psychosocial stress, and obesity are indicated as the major risk factors leading to infertility [4, 14–16].

Preclinical and clinical studies identified khat chewing as one factor for male infertility [17–20]. Khat chewing contributes to infertility by affecting spermatogenesis and plasma testosterone concentration [17, 20]. A prospective cross-sectional study on the influence of khat on seminal fluid among 214 male partners of infertile couples in Ethiopia revealed a decreased sperm count, volume, and motility in chronic khat chewers [20, 21].

A growing body of evidence points to a link between obesity or underweight and female infertility. The adipose tissue through the production of leptin, free fatty acids, and cytokines, affect both ovarian and endometrium functions in obese women [22, 23]. On the other hand, being underweight can reduce a woman’s fertility by causing hormone imbalances that affect ovulation and the chance of getting pregnant [24].

In this study, we investigated the prevalence of infertility and analyzed the socio-demographic, behavioral, and reproductive factors associated with infertility in Ethiopia to guide its prevention and treatment. Previous studies focused on women’s socio-demographic factors and failed to identify other factors, including the male partner side [25, 26].

## Methods

### Study settings and data source

Ethiopia is the second-most populous country in Africa, with one of the highest total fertility rates (4.6 children per woman) [27]. There are nine regional states and two city administrations in the country. EDHS 2016 was a nationally representative study that involved all regions and city administrations. EDHS is a household survey that uses face-to-face interviews of women aged 15 to 49 and men aged 15–59 to collect data from a wide range of demographic, health, fertility, and nutrition tracking and effect evaluation measures. The survey employs stratified, multi-stage, random sampling [27]. The variables for this study were obtained from the Couples Recode (CR file) data set. The CR data set contains data for married or living together couples who both declared that they are married (living together) and had completed individual interviews [27]. The study sample involves all married or cohabitating women aged 15–49 years.

### Description of variables

#### Dependent variables

Dependent variables were constructed based on their definition in this study (Table 1). Primary infertility is women who have been married for more than 12 months, who have had regular sexual intercourse without using contraception, and who have never conceived.

**Table 1:** Construction of dependent variables for infertility (EDHS 2016).

	Variable name in the EDHS	Label in the EDHS	Remark
Duration of a relationship more than 12 months	v852a	How long ago first had sex with your most recent partner (in months)	`
Had regular sexual intercourse in the past 12 months	v853a	Times in the last 12 months had sex with the most recent partner	More than 78 times**
No contraceptive use in the past 12 months	v361	Pattern of contraceptive use	
No birth in the past 12 months	v222	Last birth to interview in months	
Currently not pregnant	v213	Currently pregnant	
Ever had given birth	v201	Total number of children ever born	
Never conceived	v228, v201	v228 (Ever had terminated pregnancy), v201(Ever had given birth)	Never conceived: Never had terminated pregnancy and never had given birth

**Regular sexual intercourse was defined differently in several studies. It was reported that 4 to 9 times a month [28], 54 times a year [29], and 78 times [30, 31]. In the present study, more than 78 times were taken as regular sexual intercourse since it was reported specific to achieving conception.

Secondary infertility is women who have been married for more than 12 months, who have had regular sexual intercourse without using contraception, and who have at least one prior birth (EDHS 2016). We categorized women fulfilling either of the infertility definition under total infertility.

#### Independent variables

**Wealth index:** The wealth index is a composite measure of a household’s cumulative living standard. In the EDHS, households are given wealth index scores based on the number and kinds of consumer goods they own, ranging from a television to a bicycle or car, in addition to housing characteristics such as the source of drinking water, toilet facilities, and flooring materials. These scores are derived using principal component analysis. National wealth quintiles are compiled by assigning the household score to each usual household member, ranking each person in the household population by her or his score, and then dividing the distribution into five equal categories (poorest, poorer, middle, richer, and richest).

**Education level attended**: This variable indicates the level of education in the following categories; none, primary (grade 1 to 8), secondary (grade 9 to 12), and more than secondary (diploma or higher).

**Ever had terminated pregnancy:** This variable is if the respondent ever had a pregnancy that terminated in a miscarriage, abortion, or stillbirth (a pregnancy that did not result a live birth).

**Last birth a cesarean section**: This variable indicates if the last child was born by cesarean section. The base of this variable is those respondents who have had one or more births in the five years preceding the survey.

**Age at first birth:** The base of this variable is respondents who have had one or more births intending to find out the effect of teenage pregnancy on infertility.

**Body mass index (BMI)**: Is defined as a woman’s weight in kilograms divided by the square of her height in meters (W/H2). The result was then categorized based on the classification of Centers for Disease Control and Prevention (CDC), <18.5 (underweight range), 18.5 to <25 (healthy weight range), 25 to <30 (overweight range), and 30 or higher (obesity range) [32].

**Use of cigarettes or tobacco products:** This variable includes the use of cigarettes or tobacco products. The type of tobacco includes any pipe full of tobacco, chewing tobacco, snuff by nose, kreteks, cheroots or cigarillos, water pipe, snuff by mouth, betel quid with tobacco, and others.

**Khat chewing:** this variable includes chewing fresh or dried khat leaf regularly and typically within one month before the data collection period.

**Alcohol drinking:** this variable includes using any traditional or modern alcohol regularly and typically within one month before the data collection period.

### Data processing and management

We used STATA software version 14 for data processing and analysis. Before any statistical analysis, we weighted the data using sampling weight, primary sampling unit, and strata to restore the survey’s representativeness. To describe the study population, we used cross-tabulations and summary statistics.

We performed a chi-square test to check a bivariate association between infertility and each independent variable. We included all variables with a p-value <0.25 in the multiple logistic regression models. We checked the model’s goodness of fit by using Hosmer-Lemeshow statistics. We analyzed Receiver Operating Characteristics (ROC) to evaluate the predicting ability of the model (model accuracy). We presented the results using an adjusted odds ratio (AOR) with a 95% confidence interval (CI) and a p-value <0.05 as a cut-off point for declaring statistical significance.

### Ethical considerations

The Ethiopian Health and Nutrition Research Institute (EHNRI) Review Board, the National Research Ethics Review Committee (NRERC), the Institutional Review Board of ICF International, and the CDC (USA) made the ethical approval of the 2016 EDHS [33]. Moreover, we accessed the data from the DHS website (http://www.measuredhs.com) with permission on Mar 06, 2020. The accessed data was used for the registered research only and treated as confidential.

## Results

### Characteristics of respondents

#### Socio-demographic characteristics

This study analyzed a total of 6141 couples in the 2016 EDHS. The mean age of respondents was 30.4 (±SD7.8). The majorities of the respondents live in rural areas (85.2%), in the Oromia region (40.1%), and follow orthodox religion (41.2%). Sixty-one percent of women had no formal education. On the other hand, more than half of the women (51.4%) had no occupation (Table 2).

**Table 2:** Socio-demographic, behavioral, and reproductive characteristics of married women in Ethiopia, 2016 (n = 6141).

Characteristics	Frequency	Weighted percentage
Age		
<20	373	5.8
20–35	4187	68.2
>35	1581	26.0
Residence		
Urban	1393	14.8
Rural	4748	85.2
Region		
Tigray	594	5.7
Afar	357	0.7
Amhara	880	25.1
Oromia	959	40.1
Somali	499	2.5
Benishangul	562	1.1
SNNPR	857	20.9
Gambela	332	0.2
Harari	325	0.2
Addis Ababa	405	3.1
Dire Dawa	371	0.5
Religion		
Orthodox	2386	41.2
Muslim	2508	33.5
Protestant	1140	22.9
Catholic	39	0.9
Other	68	1.4
Woman’s education level		
No education	3578	61.1
Primary	1774	29.4
Secondary	481	6.0
More than secondary	308	3.5
Husband’s education level		
No education	2487	42.9
Primary	2367	41.4
Secondary	674	8.9
More than secondary	613	6.8
Wealth index		
Poorest	1575	17.7
Poorer	1040	21.2
Middle	955	20.6
Richer	953	20.5
Richest	1618	20.0
Occupation		
Not working	3208	51.4
Agricultural employee	1329	24.1
Sales	831	13.1
Skilled manual	224	3.6
Services	140	2.7
Professional/managerial	161	2.2
Others	248	2.9
BMI		
<18.5	1237	19.1
18.5-<25	4054	73.3
25-<30	525	6.3
> = 30	143	1.4
Respondent smokes cigarettes/tobacco	112	0.9
Husband smokes cigarettes/tobacco	717	7.3
Respondent drinks alcohol	2093	35.2
Husband drinks alcohol	2723	46.9
Respondent chews khat	744	14.3
Husband chews khat	2231	32.2
Age at first sexual intercourse		
<15	2592	44.7
15–19	2537	39.8
20–25	922	14.3
>25	90	1.2
Age at first birth (n = 5584)		
< = 15	895	16.7
15–19	2745	50.7
20–25	1681	28.9
25–30	211	3.2
>30	52	0.6
Ever use of contraceptive		
Ever used	3436	60.8
Never used	2705	39.2
Respondent circumcised (n = 2826)	2144	77.6
Last birth with caesarian section (n = 4335)	139	2
Ever had terminated pregnancy	668	10.2
Couple’s desire for children		
Both want same	2577	41.1
Husband wants more	1564	24.2
Husband wants fewer	401	6.5
Don’t know	1586	28.2

SNNPR, Southern Nations, Nationalities, and People’s Region BMI, Body Mass Index

#### Behavioral characteristics

Nineteen percent of the women had less than 18.5 BMI. Around 0.9% of women and 7% of their partners smoke cigarettes or tobacco products. On the other hand, 14.3% of the women and 32.2% of their partners chews khat (Table 2).

#### Reproductive characteristics

The duration of the relationship of most of the couples (75%) was more than six years, and more than one-third (39%) had never used contraception. Nearly half (50.7%) of the women had their first birth in the 15–19 age group. On the other hand, 10.2% of the women have ever-terminated pregnancies (Table 2).

### Prevalence of infertility

A total of 1487 women (24.2%; 95% CI 23.1–25.3) had infertility problems, of which the majorities were secondary (90.7%). Secondary infertility was more prevalent in the oldest age group (>35years) (16.5%), in rural residents (20.8%), in the Afar region (32.3%), in women with no formal education (23.9%), and in the poorest (25.5%). On the contrary, primary infertility was highest in the young age group (<20) (8.8%), in urban residents (3.5%), and in more educated women (4.1%) (Table 3).

**Table 3:** Socio-demographic and behavioral characteristics of women with infertility in Ethiopia, 2016 (n = 6141).

		Total infertility	Primary infertility	Secondary infertility
**Total prevalence**		24.2%, 95% CI (23.1–25.3)	2.3%, 95% CI (1.9–2.6%)	21.9%, 95% CI (20.9–23%)
**Characteristics**	**N**	**No (%)**	**No (%)**	**No (%)**
Age				
<20	373	61(12.9)	35(8.8)	26(4.1)
20–35	4187	181(12.3)	92(1.3)	769(15.7)
>35	1581	291(16.5)	11(0.9)	554(33.3)
Place of residence				
Urban	1393	271(16.6)	57(3.5)	214(13.1)
Rural	4748	1216(22.0)	81(1.3)	1135(20.8)
Region				
Tigray	594	140(22.0)	11(2.2)	129(19.8)
Afar	357	124(35.1)	11(2.8)	113(32.3)
Amhara	880	168(19.0)	19(2.3)	149(16.6)
Oromia	959	213(22.2)	12(1.3)	201(20.9)
Somali	499	132(24.6)	9(1.8)	123(22.8)
Benishangul	562	179(31.4)	14(2.8)	165(28.6)
SNNPR	857	177(21.1)	5(0.6)	172(20.4)
Gambela	332	90(24.3)	11(0.0)	79(21.5)
Harari	325	74(21.7)	12(3.5)	62(18.1)
Addis Ababa	405	69(16.4)	20(4.4)	49(12.0)
Dire Dawa	371	121(32.7)	14(3.7)	107(29.0)
Religion				
Orthodox	2386	517(20.1)	63(2.1)	454(18.1)
Catholic	39	6(15.6)	0(0.0)	6(15.6)
Protestant	1140	275(22.3)	20(1.2)	255(21.1)
Muslim	2508	669(22.2)	55(1.4)	614(20.8)
Other	68	20(37.7)	0(0.0)	20(37.7)
Education level				
No education	3578	1021(24.8)	43(0.9)	978(23.9)
Primary	1774	338(16.7)	54(2.4)	284(14.3)
Secondary	481	79(11.7)	23(3.1)	56(8.6)
Higher	308	49(13.7)	18(4.1)	31(9.6)
Wealth index				
Poorest	1575	491(26.1)	24(0.7)	467(25.5)
Poorer	1040	257(20.3)	18(1.2)	239(19.1)
Middle	955	212(20.4)	15(1.6)	197(18.8)
Richer	953	204(20.3)	20(1.6)	184(18.7)
Richest	1618	323(19.7)	61(2.9)	262(16.8)
BMI				
18.5-<25	4054	899(19.6)	86(1.6)	813(18.0)
<18.5	1237	406(29.5)	31(1.7)	375(27.8)
25-<30	525	105(16.4)	16(1.2)	89(15.2)
> = 30	143	34(20.1)	1(0.5)	33(19.6)
Respondents smoke cigarettes/tobacco	112	44(21.2)	1(0.0)	43(21.1)
Husband smokes cigarettes/tobacco	717	214(24.5)	15(2.1)	199(22.4)
Respondent drinks alcohol	2093	472(21.3)	53(2.3)	419(19.0)
Husband drinks alcohol	2723	612(20.4)	71(1.9)	541(18.5)
Respondent chews khat	744	202(24.1)	20(2.3)	182(21.8)
Husband chews khat	2231	533(20.3)	64(2.2)	469(18.1)

#### Model fitness tests

The Hosmer-Lemeshow Test’s Prob > chi2 results were 0.99, 0.82, and 0.29 for the outcome variables (primary, secondary, and total infertility), respectively. Besides, the ROC test’s areas were 0.81, 0.83, and 0.82 for primary, secondary, and total infertility, respectively. Hence, the goodness of fit tests indicated that the model assumed is correctly specified.

### Factors associated with infertility

In the binary regression, the age of women, place of residence, education level, wealth index, BMI, smoking (both partners), drinking alcohol (both partners), and khat chewing (women only) were significantly associated with infertility. Of the reproductive characteristics, the previous way of delivery had a significant association with infertility. In the multiple logistic regressions, greater than 35 years of age (AOR = 2.65), rural residence (AOR = 1.06), smoking (AOR = 2.24), and less than 18.5 BMI (AOR = 1.70) remained significantly associated with infertility (Table 4).

**Table 4:** Factors associated with infertility among couples in Ethiopia, 2016 (n = 6141).

Variables	COR(95% CI)	P-value	AOR(95% CI)	P-value
Age of respondent	** **	** **	** **	** **
<20	1.00		1.00	
20–35	1.32(0.99–1.75)	0.053	1.22(0.92–1.64)	0.16
>35	2.84(2.12–3.8)	0.00*	2.65(1.96–3.60)	0.00*
Place of residence	** **	** **	** **	** **
Urban	1.00		1.00	
Rural	1.42(1.23–1.67)	0.00*	1.06(1.01–1.39)	0.03*
Highest education level	** **	** **	** **	** **
No education	1.00		1.00	
Primary	0.59(0.51–0.68)	0.00*	0.73(0.62–0.85)	0.00*
Secondary	0.49(0.38–0.64)	0.00*	0.61(0.45–0.82)	0.00*
More than secondary	0.47(0.34–0.66)	0.00*	0.59(0.41–0.86)	0.00*
Wealth index	** **	** **	** **	** **
Poorest	1.00		1.00	
Poorer	0.72(0.59–0.89)	0.00*	0.74(0.60–0.91)	0.00*
Middle	0.63(0.51–0.77)	0.00*	0.64(0.51–0.79)	0.00*
Richer	0.60(0.49–0.74)	0.00*	0.65(0.52–0.82)	0.00*
Richest	0.55(0.46–0.66)	0.00*	0.79(0.60–1.10)	0.09
BMI	** **	** **	** **	** **
18.5-<25	1.00		1.00	
<18.5	1.71(1.5–1.96)	0.00*	1.70(1.40–2.01)	0.00*
25-<30	0.88(0.70–1.10)	0.257	0.78(0.53–1.16)	0.22
> = 30	1.09(0.73–1.63)	0.651	0,92(0.73–1.17)	0.20
Respondent smokes cigarettes/tobacco	2.06(1.43–2.96)	0.00*	2.24(1.35–3.44)	0.00*
Husband smokes cigarettes/tobacco	1.39(1.17–1.65)	0.00*	1.08(0.88–1.32)	0.43
Respondent drinks alcohol	0.87(0.75–1.00)	0.03*	0.92(0.76–1.10)	0.38
Husband drinks alcohol	0.86(0.75–0,98)	0.01*	0.93(0.78–1.12)	0.48
Respondent chews khat	1.19(0.98–1.46)	0.046*	1.15(0.93–1.43)	0.18
Ever use of contraceptive	0.92(0.80–1.06)	0.26	-	-
Respondent circumcised ^a^	1.24(1.001–1.52)	0.043*	-	-

**Note:** COR, crude odds ratio; AOR, adjusted odds ratio

*, significant association; CI, confidence interval; BMI, body mass index

^a^, not included in the multiple logistic model due to a large number of missing values (n = 3315).

We conducted a sub-analysis of factors associated with primary and secondary infertility. In the multiple logistic regression, primary infertility was significantly higher among women whose partners chew khat (AOR = 1.62) and drink alcohol (AOR = 1.55) (Table 5). On the other hand, secondary infertility was significantly higher among women >35 years of age (AOR = 3.58), less than 18.5 BMI (AOR = 1.59), >30 BMI (AOR = 1.54), smokers (AOR = 1.85) and, ≤15 age at first birth (AOR = 1.40) (Table 6).

**Table 5:** Factors associated with primary infertility among couples in Ethiopia, 2016 (n = 6141).

Variables	COR (95%CI)	P-value	AOR (95%CI)	P-value
Age				
<20	1.00		1.00	
20–35	0.21(0.14–0.33)	0.000*	0.28(0.15–0.50)	0.00*
>35	0.06(0.03–0.13)	0.000*	0.13(0.04–0.39)	0.00*
Place of residence	** **		** **	
Urban	1.00		1.00	
Rural	0.41(0.29–0.57)	0.000*	(0.31–1.39)	0.28
BMI	** **		** **	
18.5-<25	1.00		1.00	
<18.5	1.19(0.78–1.80)	0.425	1.36(0.87–2.11)	0.17
25- <30	1.45(0.85–2.47)	0.170	0.98(0.53–1.89)	0.96
> = 30	0.32(0.04–2.37)	0.267	0.18(0.02–1.33)	0.09
Highest education level				
No education	1.00		1.00	
Primary	2.6(1.72–3.87)	0.000	1.2(0.77–1.89)	0.40
Secondary	4.1(2.47–6.91)	0.000	1.6(0.87–3.07)	0.12
More than secondary	5.1(2.91–8.96)	0.000	1.7(0.82–3.49)	0.15
Wealth index				
Poorest	1.00		1.00	
Poorer	1.1(0.61–2.11)	0.681	1.11(0.48–2.13)	0.75
Middle	1.03(0.54–1.98)	0.926	1.01(0.77–2.87)	0.96
Richer	1.34(0.76–2.52	0.286	1.49(0.80–2.96)	0.23
Richest	2.5(1.57–4.08)	0000*	1.49(0.66–3.37)	0.33
Husband smokes cigarettes/tobacco				
No	1.00			
Yes	0.92(0.53–1.60)	0.769	Na	Na
Respondent drinks alcohol				
No	1.00			
Yes	1.21(0.85–1.73)	0.290	Na	Na
Husband drinks alcohol				
No	1.00		1.00	
Yes	1.29(0.92–1.82)	0.140	1.55(1.06–2.28)	0.02*
Respondent chews khat				
No	1.00			
Yes	1.24(0.78–1.97)	0.370	Na	Na
Husband chews khat				
No	1.00		1.00	
Yes	1.49(1.05–2.09)	0.024*	1.62(1.12–2.36)	0.01*
Age at first sexual intercourse				
< = 15	1.00		1.00	
15–19	1.47(0.97–2.22)	0.073	0.97(0.63–1.51)	0.92
20–25	2.44(1.53–3.89)	0.000*	2.27(1.35–3.81)	0.00*
>25	5.38(2.36–12.28)	0.000*	5.93(2.3–15.2)	0.00*

**Note:** COR, crude odds ratio; AOR, adjusted odds ratio

*, significant association; CI, confidence interval; BMI, body mass index; Na, not applicable

**Table 6:** Factors associated with secondary infertility among couples in Ethiopia, 2016 (n = 6141).

Variables	COR(95% CI)	P-value	AOR(95% CI)	P-value
Age of respondent				
<20	1.00		1.00	
20–35	1.82(1.20–2.77)	0.005*	1.54(1.03–2.33)	0.037*
>35	2.89(1.92–4.34)	0.000*	3.58(2.34–5.48)	0.000*
Place of residence				
Urban	1.00		1.00	
Rural	1.73(1.45–2.07)	0.000*	1.11(0.82–1.49)	0.499
Highest education level				
No education	1.00		1.00	
Primary	0.51(0.44–0.58)	0.000*	0.74(0.63–0.87)	0.000*
Secondary	0.35(0.26–0.47)	0.000*	0.54(0.39–0.76)	0.000*
More than secondary	0.29(0.19–0.45)	0.000*	0.53(0.33–0.85)	0.009*
Wealth index				
Poorest	1.00		1.00	
Poorer	0.71(0.58–0.87)	0.001*	0.73(0.59–0.91)	0.004*
Middle	0.62(0.50–0.76)	0.000*	0.63(0.51–0.79)	0.000*
Richer	0.57(0.46–0.70)	0.000*	0.61(0.48–0.77)	0.000*
Richest	0.46(0.38–0.55)	0.000*	0.76(0.57–1.03)	0.079
BMI				
18.5-<25	1.00		1.00	
<18.5	1.73(1.51–1.99)	0.000*	1.59(1.37–1.84)	0.000*
25- <30	0.81(0.64–1.04)	0.102	0.92(0.71–1.21)	0.561
> = 30	1.20(0.79–1.80)	0.390	1.54(1.01–2.35)	0.048*
Respondent smokes cigarettes/tobacco	2.25(1.56–3.25)	0.000*	1.85(1.21–2.82)	0.004*
Husband smokes cigarettes/tobacco	1.43(1.20–1.70)	0.000*	1.07(0.87–1.32)	0.503
Respondent drink alcohol	0.84(0.72–0.98)	0.030*	0.89(0.74–1.08)	0.246
Husband drinks alcohol	0.82(0.71–0.95)	0.008*	0.90(0.75–1.08)	0.272
Respondent chew khat	1.17(0.95–1.44)	0.392	-	-
Husband chew khat	0.94(0.81–1.10)	0.392	-	-
Age at first birth				
20–25	1.00		1.00	
< = 15	1.50(1.25–1.80)	0.000*	1.40(1.15–1.69)	0.001*
15–19	1.22(1.05–1.40)	0.007*	1.21(1.03–1.41)	0.017*
25–30	0.98(0.67–1.43)	0.905	0.88(0.58–1.33)	0.553
>30	0.99(0.533–1.85)	0.987	1.12(0.56–2.22)	0.749
Ever had terminated pregnancy	1.25(1.04–1.52)	0.020*	1.20(0.97–1.47)	0.090
Respondent circumcised ^a^	1.28(1.005–1.62)	0.046*	-	-
Last birth a caesarian-section ^b^	0.42(0.24–0.74)	0.003*	-	-

**Note:** COR, crude odds ratio; AOR, adjusted odds ratio

*, significant association; CI, confidence interval; BMI, body mass index; Na, not applicable

^a^ not included in the multiple logistic model due to a large number of missing values(a = 3315 missing values; b = 1806 missing values)

## Discussion

In this study, we assessed the prevalence of infertility and its associated factors among couples in Ethiopia using the 2016 EDHS data.

More than twenty percent of couples reported having infertility in the 12 months. Abnormal BMI, smoking, khat chewing, alcohol drinking, and young age at first birth were significantly associated with infertility.

The prevalence of infertility in the current study was lower than in the 2013 DHS in Nigeria (31.1%). In Nigeria, the prevalence of primary and secondary infertility was 17.4% and 34.1%, respectively [34]. The variation in the results could be due to methodological differences. This study used a constructed approach to estimate the prevalence of infertility, while the study in Nigeria used the current duration approach.

The prevalence of infertility in the present study was much higher than in a study in the United States that used both the current duration (15.5%) and the constructed approach (7.0%) to estimate the magnitude of infertility [35] and a prospective study on 2151 couples from two counties of Shanxi Province in northern China (13.6%) [13]. The variation in the magnitude of infertility could be due to the differences in the availability of infertility care, sociocultural value surrounding infertility, and the study design differences [36].

In the present study, being underweight was significantly associated with infertility. This finding is in line with a study in Northern China [37]. The underlying mechanism for the association of underweight and infertility could be because low body weight results in functional hypothalamic failure, ovulatory menstrual cycles, and amenorrhea [38]. Decreased adipose tissue in underweight women metabolizes estrogen into a less potent form [39].

The present study revealed the association of obesity with infertility. This finding was in agreement with a prospective study in China [13], a case-control study in Bangladesh [40], and a systematic review of several studies [41]. Studies revealed obesity contributes to anovulation, menstrual irregularities, reduced conception rate, and a reduced response to fertility treatment. It also increases miscarriage and contributes to maternal and perinatal complications [22–24, 42].

The current study revealed a significant association between older age of women and infertility. This could be due to the lower number of eggs and abnormal chromosomes in older women. Moreover, older women are at higher risk of uterine fibroids, endometriosis, and pelvic infection [43–45].

In the present study, the use of cigarettes/tobacco was significantly associated with infertility (AOR = 2.29). A case-control study in Iran (AOR = 2.88) [46], a Behavioral Risk Factor Surveillance System (BRFSS) conducted by the CDC (AOR = 1.98) [47], and a National Survey of Family Growth (NSFG) in the USA (AOR = 1.77) [48] reported similar findings. The association between smoking and infertility could be because the chemicals in the substance cause fewer eggs in the ovary and lower quality eggs. It even causes irreversible ovarian damage over a long period of use. Moreover, studies indicated that anti-mullerian hormone levels are lower, and the onset of menopause is about five years sooner in women who smoke [49–51].

In the current study, women whose partners chew khat had significantly higher odds of primary infertility (AOR = 1.55). A study on factors associated with time-to-pregnancy on 1150 pregnant women in Addis Ababa revealed a similar result (AOR 1.66) [19]. This finding could be because khat chewing affects the quality of sperm, lowers libido, and affects the potency of male sexuality by affecting spermatogenesis and plasma testosterone concentration [17, 18].

In the present study, women whose partners drink alcohol had significantly higher odds of primary infertility. The reason for this could be alcohol drinking leads to atrophy of the testes, impotence, reduced libido, and worse quality of sperm [52–55]. Hence, the American Society for Reproductive Medicine (ASRM) recommends that couples should avoid excessive alcohol drinking (≥ 2 drinks a day) during attempts to conceive [51].

The present study revealed that a higher level of education has a negative association with infertility. This could be due to educated people being more aware of the treatment, and complications, leading to an increased probability of seeking help [56, 57].

The present study found that a high wealth index has a protective effect on infertility. The reason for the finding could be a good economic status can resolve infertility problems by encouraging people to have fast access to treatments [56, 58]. Moreover, a better economy would influence people’s decision to have children [59].

The present study revealed a significant association between lower age at first birth and secondary infertility. Several studies indicated that teenage pregnancy is associated with adverse outcomes such as obstructed labor, pre-eclampsia, anemia, operative deliveries, puerperal endometritis, and postpartum hemorrhage, which affect fertility in women [60–63].

### Strengths and limitations of the study

The study tried to estimate the national prevalence of infertility by constructing variables from DHS data. Besides, the study assessed the behavioral factors contributing to infertility from the male partner’s side. However, given the cross-sectional design, the temporal nature of factors associated with infertility needs to be considered. In addition, the implicit assumption that those not at risk of pregnancy (e.g., using contraception) are fertile and unrecognized pregnancies may have biased our findings. Furthermore, we restricted the study sample to cohabitating couples, which might result in the exclusion of women no longer in a relationship being responsible for infertility.

## Conclusions

A significant percentage of couples in Ethiopia are struggling with infertility. Both female and male-related factors have a substantial role in infertility. Primary infertility was significantly higher in women with partners who chew khat and drink alcohol. Secondary infertility was significantly associated with maternal under-nutrition, obesity, smoking, and young age at first birth. This study also found a negative association between women’s socio-demographics (education, wealth index) with infertility. The findings of this study imply taking action on preventable factors is a critical strategy to prevent both primary and secondary infertility and will improve the health status of the couples in other ways. As a result, emphasis should be placed on health information dissemination and raising awareness of the preventable factors of infertility. Furthermore, improvement of the economy and level of education are the suggested strategies to prevent infertility.

## Abbreviations

BMI: Body Mass Index
CHMS: College of Health and Medical Sciences
CR: Couples Recode
DHS: Demographic and Health Survey
EDHS: Ethiopian Demographic and Health Survey
NSFG: National Survey of Family Growth
